# TreeFam v9: a new website, more species and orthology-on-the-fly

**DOI:** 10.1093/nar/gkt1055

**Published:** 2013-11-03

**Authors:** Fabian Schreiber, Mateus Patricio, Matthieu Muffato, Miguel Pignatelli, Alex Bateman

**Affiliations:** ^1^Wellcome Trust Sanger Institute, Wellcome Trust Genome Campus, Hinxton, Cambridgeshire CB10 1SA, UK and ^2^European Molecular Biology Laboratory, European Bioinformatics Institute (EMBL-EBI), Wellcome Trust Genome Campus, Hinxton, Cambridge CB10 1SD, UK

## Abstract

TreeFam (http://www.treefam.org) is a database of phylogenetic trees inferred from animal genomes. For every TreeFam family we provide homology predictions together with the evolutionary history of the genes. Here we describe an update of the TreeFam database. The TreeFam project was resurrected in 2012 and has seen two releases since. The latest release (TreeFam 9) was made available in March 2013. It has orthology predictions and gene trees for 109 species in 15 736 families covering ∼2.2 million sequences. With release 9 we made modifications to our production pipeline and redesigned our website with improved gene tree visualizations and Wikipedia integration. Furthermore, we now provide an HMM-based sequence search that places a user-provided protein sequence into a TreeFam gene tree and provides quick orthology prediction. The tool uses Mafft and RAxML for the fast insertion into a reference alignment and tree, respectively. Besides the aforementioned technical improvements, we present a new approach to visualize gene trees and alternative displays that focuses on showing homology information from a species tree point of view. From release 9 onwards, TreeFam is now hosted at the EBI.

## INTRODUCTION

With the increasing availability of new genome sequences, the task of mapping genes to their corresponding counterpart in other organisms becomes even more important. In this context, orthology—two genes in different organisms that are the result of a speciation event—is often used to find these corresponding genes and to allow transfer of annotation from the known to the unknown gene.

The original orthology definition of Fitch used a phylogenetic tree to determine orthology (1). Some of the orthology databases available today follow the same principle and predict orthology/paralogy relationships based on phylogenetic trees reconstructed from alignments of homologous sequences. One of these databases is TreeFam, which aims at providing comprehensive gene evolution information and orthology assignments for animal gene families (2,3). Other tree-based databases that differ either in taxonomic scope and/or tools used are Panther (4), Ensembl (5), Phylofacts (6), PhIGs (7) and PhylomeDB (8). Besides tree-based databases, there are plenty of graph-based tools for the routine generation of genome-wide orthology mappings, e.g. Hieranoid (9), OMA (10), OrthoDB (11) or OrthoMCL (12). While graph-based approaches are generally computationally less expensive, the results of tree-based methods can be easily visualized and are more informative in certain situations, as gene losses/duplications can be inferred and dated on a phylogenetic tree. While most of the orthology prediction methods mentioned above use their own pipeline to build data sets and make releases, TreeFam and Ensembl have a long history of successfully sharing large parts of their production pipeline.

In this update, we describe the latest release 9 of TreeFam where we have increased the number of species from 79 to 109 (104 fully sequenced animal genomes + 5 outgroup species). The TreeFam project was in stasis for some time, when we revived it we gave the website a facelift. This together with some new features to visualize gene family trees and to allow on-the-fly-orthology prediction for a user-supplied sequence make TreeFam an even more useful resource for studying animal gene families and orthologs and paralogs in animal genomes.

## MATERIALS AND METHODS

### Sequence data

TreeFam 9 covers 109 fully sequenced genomes from 104 animal species and the following 5 outgroups species: the two choanoflagellates *Monosiga brevicollis*, *Proterospongia sp.*, the Baker's yeast *Saccharomyces cerevisiae,* the fission yeast *Schizosaccharomyces pombe* and the cress *Arabidopsis thaliana*. This number represents an increase of 30 species over TreeFam 8. TreeFam 9 covers all species from Ensembl v69 and Ensembl Metazoa 15, plus two Choanoflagellates (*M. brevicollis*, *Proterospongia sp.*) from JGI (http://genome.jgi-psf.org) and the pine wood nematode *Bursaphelenchus xylophilus*, the entomopathogenic nematode *Heterorhabditis bacteriophora*, the rat parasite *Strongyloides ratti* and the Northern root-knot nematode *Meloidogyne hapla* from Wormbase (13). For TreeFam 9, all sequences were downloaded in December 2012.

### Overall strategy

TreeFam and Ensembl have been long-standing collaborators on their production pipeline. Until recently, this mostly covered the gene-tree building and orthology assignment steps. With TreeFam 9, TreeFam has fully adopted the Ensembl Compara pipeline with the exception of how gene families are defined. While Ensembl Compara rebuilds its gene families for every release running an all-versus-all Blast (14) search and clustering the proteins with hcluster_sg, TreeFam uses an HMM-based approach that guarantees that TreeFam families are stable over time. Based on a set of HMMs from the previous TreeFam release, new sequences are assigned to existing families using HMMER 2.3 (15). For each gene family, alignments are built with either MCoffee (16) for families with <200 members or Mafft (17) otherwise. Alignments are then filtered to include conserved positions as described in (2). For each family, we build a set of gene trees using TreeBest as described in (3). TreeBest builds five gene trees based on the amino acid and a back-translated codon alignment. The five trees are then merged into one consensus tree using a species tree as a reference (18). The advantage of using amino acid and codon sequence information is that trees based on the former are more suitable to resolve distant relationships, whereas trees based on the latter are more suitable for close relationships. The resulting consensus tree is reconciled with the NCBI taxonomy tree (19) using the ‘Duplication/Loss Inference’ algorithm (2,18).

### New website

As the TreeFam project was put on hold for a few years, we figured it might be the best time to give the website a facelift. The layout of the new TreeFam website is adopted from that of the Pfam [(20), http://www.pfam.sanger.ac.uk] and Rfam database [(21), http://www.rfam.sanger.ac.uk]. The new TreeFam website provides users with a familiar design that makes it easier to understand how the database is structured. We also implemented an HMM-based sequence search using HMMER. This allows users to search a protein sequence against our set of 15 753 TreeFam families (http://www.treefam.org). Furthermore, we focussed on implementing the following two features on our website:

### Gene tree visualization

**Figure 1.**
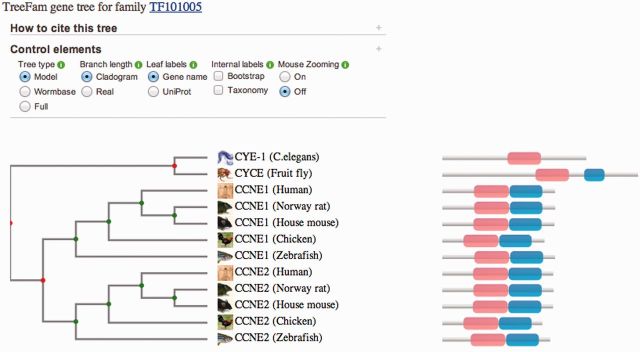
This figure shows the pruned tree for TreeFam family TF101005 (Cyclin E) showing model species only. Alongside the leaves protein domain information is plotted. The top panel allows to select/deselect various tree annotations features, e.g. bootstrap values, internal node names. The panel on the right hand side show information on mouse over for sequences and protein domains.

Due to their potentially complex history, the visualization of the evolution of gene families can be a daunting task. Given the 109 species in TreeFam, it can take some time to interpret gene trees of even single copy gene families, let alone families with lots of duplications and losses. To make the interpretation of our trees easier we developed a new gene tree visualization widget based on Javascript and the D3 library (http://www.d3js.org). This allows us to provide interactive trees and make their interpretation easier. As the user might not be familiar with some of the species used in TreeFam, we show a pruned gene tree of model species (as defined by http://geneontology.org/GO.refgenome.shtml) by default. The gene tree visualization also incorporates Pfam protein domain information for easier interpretation of the evolutionary history of the gene (see Figure 1 for an example).

### Orthology-on-the-fly

The identification of orthologs in related organism is a routine task and many databases/tools are available to do that (see ‘Introduction’ section). Some of the databases can be installed locally, which is not ideal in cases where the target is to find orthologs for a single/few genes only. To fill this gap, we developed a quick orthology-on-the-fly prediction tool that is built on top of the HMMER search mentioned before. In our HMMER search page, users can tick the box ‘insert into tree’ to not only get hits in TreeFam (additionally we search Pfam for protein domain hits) but additionally align the user-supplied sequence to the best matching TreeFam family using MAFFT (17) and then adding the sequence into the corresponding gene tree using the family gene tree as a reference for the RAxML-EPA algorithm (22).

We benchmarked different evolutionary placement algorithms for inserting a sequence into a reference tree. Note that rebuilding the tree is in most cases computationally too complex to be offered as a web service. We tested RAxML-EPA algorithm (22) and Pagan (23) on a subset of TreeFam families and used the gene tree build with TreeBest as a reference. We were interested in accuracy and run time, so we looked at the average scaled Robinson-foulds distance (24) defined as:

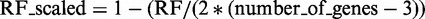



For the run time, we took the average run time per job in seconds. The lower the average distance between one of the methods and TreeBest, the better it performs. The results (see Table 1) indicated that RAxML offered the best results in accuracy (ML-option) and run time (parsimony-option).

**Table 1.** This table shows in the first column the tested tools Pagan, RAxML-ML and RAxML-MP, in the second column the average Robinson-Foulds distance between trees generated with each of the three tools and the gene tree generated by TreeBest used as a reference

Program	Average RF distance	Runtime (s)
Pagan	0.267	84.502
RAxML-EPA-ML	0.295	66.178
RAxML-EPA-parsimony	0.206	0.164

The last column shows the average runtime for each tool.

On completion of the orthology-on-the-fly search, TreeFam provides a summary page of the best-matching TreeFam family and a gene tree with the newly added sequence. Given that all our gene trees have protein domain annotations, we search the Pfam database to report Pfam protein domain matches on the sequence, making it easy to spot differences in the domain architecture of the new sequence and members of the gene family.

### Using TreeFam

We provide a wide range of ways for the user to query TreeFam, e.g. to query for a gene of interest, we provide searches by accession number, keyword and several external database ID (e.g. UniProt, HGNC). The user can also use the new HMMSearch and the Orthology-on-the fly searches. All the data can be freely downloaded from http://www.treefam.org/download. This includes families, alignments, HMMs, trees, homologs and mappings to external databases. Links to TreeFam families are present in many well-known databases, e.g. Pfam, HGNC, Wormbase, GeneCards, Ensembl.

## DISCUSSION AND FUTURE PLANS

We have successfully revived the TreeFam project, made a new release with a new website available and added some interesting features such as the orthology-on-the-fly option, which allows researcher, e.g. ones doing gene annotation to verify their gene predictions. Furthermore, we provide a set of HMMs of single-copy genes across animals that could be used to check gene predictions as well.

In the future, we will work on increasing the gene coverage. One approach is to use the all-versus-all Blast approach used by Ensembl Compara to rebuild all families and add new families that do not overlap with any existing family. In addition we will continue to improve the quality of the gene trees, improve our user interface and increase the number of species available in TreeFam.

## FUNDING

Wellcome Trust [WT077044/Z/05/Z]; The Wellcome Trust [WT098051]; BBSRC [BB/I025506/1]; European Molecular Biology Laboratory (to M.M. and M.P.); European Community's Seventh Framework Programme (FP7/2007-2013) [222664]. (‘Quantomics’). Funding for open access charge: Wellcome Trust [WT077044/Z/05/Z].

*Conflict of interest statement*. None declared.
